# Fostering cultural resilience: assessing the success of a locally engaged and adapted mental health intervention in Gaza

**DOI:** 10.3389/fpubh.2024.1390211

**Published:** 2024-07-16

**Authors:** Sarah Rockowitz, Rasha Bayoumi, Nora Parr, Abdullah Awad, Mohamed Altawil, Khalifa Elmusharaf

**Affiliations:** ^1^School of Psychology, University of Birmingham, Birmingham, United Kingdom; ^2^School of Psychology, University of Birmingham Dubai, Dubai, United Arab Emirates; ^3^School of English, Drama, and Creative Studies, University of Birmingham, Birmingham, United Kingdom; ^4^Institute for Critical Thought, Amman, Jordan; ^5^School of Life and Medical Sciences, University of Hertfordshire, Hatfield, United Kingdom; ^6^School of Public Health, University of Birmingham Dubai, Dubai, United Arab Emirates

**Keywords:** Palestine, science diplomacy, PTSD, cultural competence, trauma

## Abstract

In ongoing-conflict-affected regions like Gaza, the prevalence of complex and intersecting post-traumatic stress disorders (PTSDs) necessitates innovative interventions. Our study explores a mental health care approach that has been culturally adapted for 15 years to address the complex landscape of PTSD in the Gaza Strip. Tarkiz was initially developed as ‘Focusing’, a metacognitive approach founded by Eugene Gendlin in 1950s Chicago. Tarkiz has been iteratively adapted and implemented for over a decade in Gaza by a team of local practitioners. The program’s unique emphasis lies in its engagement approach, which relies on community participation and partnership building. The aim of this study was to qualitatively explore the perceived success of the program from the perspectives of the practitioners who adapted and delivered the program and the clients who participated in it. Data collection was driven by a multicultural research team, and prioritized capacity-building opportunities for Palestinian practitioners who lead on the development of research questions defining success. This exemplifies a successful science diplomacy model, emphasizing a collaborative approach, cultural sensitivity, and adaptable partnerships essential in global public health.

## Introduction

While a great deal of work has been done on the cultural adaptation of trauma interventions, a significant portion of this research focuses on trauma from the lens of PTSD; these assume that trauma stems from a specific incident. Findings from research on cultural adaptation indicate that there are multiple frameworks that can be used to adapt various forms of therapies to different cultural contexts, but that a ‘one size fits all’ approach is often not appropriate, as individuals from certain cultures may not benefit from an approach that individuals in a different cultural context would find beneficial (1, 2). Work in the humanities has also long critiqued the idea of a single definition of trauma (3), suggesting instead that what constitutes trauma in different locations, and what defines reactions to these experiences is certainly culturally and historically dependent (4). Work in Global Mental Health suggests that when designing an intervention, it is important to think not only of which empirically supported treatments may address the issues, but also how the target group typically handles trauma regardless of external interventions (2).

In ongoing-conflict-affected regions like Gaza, the prevalence of what have been best described as complex and intersecting PTSDs necessitates innovative work; not just to assist in addressing mental health issues but also in broadening the definitions of psychological harm and its redress. In Gaza, the community and practitioners are collectively living in a context of continuous and overlapping events, systems, and structures of harm. Where most PTSD-informed interventions presume the end of trauma and seek a safe space for recovery, but practitioners can assume no such safety, and must generally presume a continuation of trauma. Multiple issues raised in the broader research are relevant in the Gazan context, where trust of international interventions is limited, the stigma of mental health treatment (or rather the existence of a mental health problem) is considerable, and where the landscape of international funding means it is exceptionally difficult to sustain programming long enough to offer grassroots solutions (5, 6). There are also limited opportunities for the long-term development of local solutions through education systems, as local training opportunities are extremely limited, and ongoing processes of siege and occupation hamper the development and sustainability of locally run programs.

This paper looks at one long-standing locally run mental health initiative at the Palestine Trauma Center. The Palestine Trauma Center (PTC) is a small non-governmental organization in Nuseirat, an area of north-central Gaza. PTC was founded by Dr. Mohamed Altawil, who was born and raised in the area, to respond to the gaps in mental health provision in the region. Dr. Altawil undertook a PhD in the UK at the University of Hertfordshire, and based on his findings, a group of professionals were brought together in 2007 to form PTC (Gaza). This organization helped train therapists in mental health skills and acted as a referral center for those with mental health disorders and PTSD symptoms. The support group, PTC (UK) was set up in 2010. This formalized the relationship between the center in Gaza and a board in the UK that sought to support the work through regular meetings with staff and fundraising efforts. Dr. Altawil and the center’s governing board are based in the UK. The organization has faced multiple challenges and is built by local residents to address the situation in Gaza, meaning that the center recognizes need, has been developed to fit local requirements, operates on a first-come first-served basis, is free to access, and works to reduce stigma. The UK board works to support these aims.

Given this context, assessing why a long-term adaptation of a program is understood as effective is crucial. Not only does this work provide a renewed set of questions with which to assess the potential effectiveness of other programs, but through further evaluation of interviews with both clients and practitioners, a 360-degree model can be generated of the meaning of mental health and what it means to support it. Crucially, for this project, an assessment of the perceived effectiveness of mental health programming included consultation with practitioners and clients about the definition of success itself.

## Literature review

When this data was collected in 2022, the landscape of mental health provision in non-Western contexts had reached several overlapping consensuses. First, that western-rooted notions of PTSD and their underlying cultural assumptions about the nature of trauma and its effects problematically dominated the field (7). Second, the most striking assumption about PTSD-informed interventions was that trauma was the response to a single event that disrupts a ‘normal’ and healthy life (3). Global mental health and postcolonial trauma theory called for a revised approach that understands the longevity of trauma and complex contexts of mental health beyond the Euro American model (8). Scholars have shown how existing tools of PTSD-informed mental health evaluation are insufficient for capturing the types, kinds, and magnitudes of non-Western (or non-normative) experiences of harm and demonstrated that existing methods of description fail to adequately assess diverse mental health conditions (4, 9).

PTSD-informed programs that do intervene in vulnerable communities, especially in the Global South, have been criticized for often doing more harm than good (10). They have, for example, excluded individuals from accessing help, for example when externally defined symptoms are not displayed, or the experience of harm is articulated in ways that do not register on existing diagnostic checklists (1). These programs have also failed to be appropriately culturally adapted, have problematically interpreted ‘recovery,’ and have included normative assumptions about race/class/other factors that exclude non-Western experiences (11–13). While programs like Stress First Aid or Psychological First Aid, which were developed to help those in high-stress occupations which may increase vulnerabilities, are said to have been effective in the context of the pandemic (and were developed with mechanisms of some basic cultural adaptation), they are both limited in depth and scope (14). As ‘first aid’ these programs also appear to assume the possibility or at least necessity of follow-up protocols, which would of course in turn need to account for the ongoing nature of harm (14).

There are many examples of how design and adaptation might account for local culture and frameworks of experience. Western cultures are often individualistic, and thus mental health interventions are designed with that in mind. Other societies, however, are more collectivist, which must be considered when designing an intervention. This was true of a mental health program targeted at Karen refugees from Myanmar, wherein the researchers understood that Western PTSD interventions that often focus on the independent self and pursuing individual goals would not be applicable to a group that views oneself in connection with others and prioritizes fulfilling social obligations (15). The PTSD intervention in that study was designed with connectedness and communion in mind and was largely acceptable by the intervention participants (*IBID*). Conversely, after the 2004 tsunami that killed tens of thousands of people in Sri Lanka, Western mental health professionals flocked to Sri Lanka to offer their services without doing any research on how Sri Lankans typically dealt with trauma. As a result, thousands of Sri Lankans were offered services and informed about what the state of their mental health should be according to Western standards, which caused the emergence of problems that had not been seen before in Sri Lanka and arguably did more harm than good (16). These examples, and countless others, emphasize the importance of culturally adapted PTSD interventions, although even when existing interventions are adapted, critical elements may still be missed.

Within this context, science diplomacy and its approaches offer critical tools to transcend current political and disciplinary boundaries. At its core, science diplomacy emphasizes collaboration, cross-cultural understanding, and the co-creation of solutions. It offers an adaptive approach, acknowledging that effective interventions must be contextually relevant and collaboratively developed. In the context of mental health interventions in Gaza, science diplomacy offers ways of seeing context and approaching research that are as much tools of listening as they are of amplifying knowledge that is usually ignored. Science diplomacy fosters partnerships and mutual understanding and bridges the gap between global mental health knowledge and the specific needs of the Gazan population.

In the case of mental health interventions in Gaza, science diplomacy is not just a theoretical concept but a practical imperative. It enables the recognition of programs like Tarkiz, which stands as a testament to the effectiveness of culturally sensitive, community-driven approaches, long term adaptation, and respect for local knowledge. It is only through the tools of science diplomacy and the work of postcolonial/decolonial studies that we can access as knowledge the work of adaptation. As we present the findings made possible through this approach, we unravel its potential to enhance the resilience of communities by fostering interventions that are not only scientifically sound but also deeply embedded in the cultural and social realities of the population they aim to serve.

## The Palestine trauma center

The services that PTC offers are specifically designed to address the mental health needs of the community structurally, functionally, and ideologically. Services are free and are offered on a first-come-first-served basis. There are also services for all ages and stages, including family-focused programs, and all programs are promoted and amplified through a strong community network. This model not only addresses the financial and political instability of the Gaza Strip, but also the reality of extreme poverty that the PTC community exists within. Free services reduce the financial barrier to mental health care and the PTC believes this ease of access also helps reduce the stigma associated with mental health services. The issue of stigma is also addressed through the referrals system (between trusted organizations) and strong community support. The center is constantly inundated with community members and referrals from the local Ministry of Health, schools, and other NGOs, and has developed systems to manage the high number of potential clients as well as the existing deficiencies in diagnostic criteria (so evaluation does not prioritize or de-prioritize an individual). While the center does ‘score’ individuals on an adapted PTSD score sheet, this does not determine whether an individual is able to access the program. The community support and amplification is essential because there is no consistent government infrastructure in Gaza, and medical resources are already overtaxed, so there is little reliable and affordable mental health support. As political conflict has hampered efforts at providing already underfunded services, all the center’s expenses are paid for by donations, with the occasional addition of small sources of funding related to research projects such as the present study (17).

The PTC model is a successful one, is one of a small handful of mental health organizations in Gaza, the most widely known of which is the Gaza Community Mental Health Program, which is also the only NGO to offer regular clinical services in Gaza. Because of a shortage of trained psychiatrists and psychotherapists, most other NGOs like PTC offer services developed by professionals and run by local workers who receive appropriate training to facilitate the programs, often with backgrounds in social work and community organizing (18). This model has several advantages in that it does not rely on training and licensing procedures currently unavailable in Gaza, which are difficult to access otherwise given imposed restrictions on travel. It also allows for the provision of relatively reliable mental health services, which is key for not only the long-term development of programs, but also for establishing trust within the community (19).

The set of programs that PTC offers to its community has also been honed to balance need and capacity. They work both independently and in complement to engage the community, normalize discussions of mental health, and provide essential tools for psychological wellbeing in a context of harm. In addition to Tarkiz, PTC offers programs geared toward children, families, and teens. These include the Family Therapy Program, which takes the family as a unit of analysis while still focusing on individual mental health; the Psycho-Social Support Program, which tends to the social context within which psychological symptoms appear, and Community Wellness Focusing, which focuses on the irreducible role of the community in the practice of building resistance (20). They also offer programming for children, including the Friday of Joy initiative, which brings psychosocial support and community making into the streets, reclaiming spaces of violence, often where bombings or bombardments have created open spaces in the dense urban infrastructure. Friday programs, which include plays, minor circus acts, and pantomimes work to destigmatize issues around mental health, and share wellness solutions with children and their families. All of these services are always free to access.

## Tarkiz

Tarkiz is an adaptation and translation of a theory and method devised by the American philosopher Eugene Gendlin in the 1950s. ‘Focusing,’ as Gendlin developed it, puts emphasis on recognizing personal and individual responses to harm or harmful situations and understanding reactions to these (21). The therapeutic approach addresses how the individual has experienced harm (rather than the nature of the harm itself). This approach allows PTC and its community to work on their own largely unarticulated definition of harm and bypass the restrictive and occluding definitions that research has identified as problematic in PTSD-based therapeutic approaches. After being personally impressed by the Focusing technique, Altawil adapted and tested it with Arab students in Hertfordshire during his PhD work, then returned home to Gaza in 2010 to initiate the first training course. Since then, he has worked with practitioners to continue fine-tuning the program based on client feedback and staff reflection. Early study by Altawil (20) already found that the Focusing program significantly reduced reported PTSD indicators. Even after these findings, however, the center continued to refine the service. Adaptation has been ongoing since 2010 so that techniques, methods, specific activities, materials, and even locations have (and continue to be) changed to suit the needs of the community.

As PTC practitioners describe it:

“The Tarkiz program for psychological and social support aims to enable participants to get to know themselves, communicate with themselves effectively, compassionately and without blame, and then deal consciously with others in an effective way.”

The program is delivered over 12 sessions. These are held roughly every week, either at the PTC headquarters or in facilities of a network of community organizations in order to make the material accessible to wider audiences. Groups are small (ten or less), and they are run by trained PTC practitioners. Tarkiz is run on a 1:1 basis occasionally, depending on availability of staff. All data here is from group participants. Over the past five years, participants were also included in a WhatsApp group, which helps communicate amongst the almost inevitable time and date changes due to blackouts, air strikes, and other matters that necessitate a change. During the first covid lockdown, PTC developed a manual so that clients participating online could follow along, and the manual continued to be used (with activity cards to cut out, and example illustrations, assessment tools and background information on the sessions and techniques) when printing was available. The sessions introduce the concept of ‘focusing’ and its emphasis on developing good listening skills that are applied to both the individual and their peers/community.

After establishing a basis of trust and an agreement from participants that information will be confidential and the session is a space to share openly, the following sessions work through material on what it means to listen to others (2), developing a safe space to recall in times of stress (5), explaining the purpose of ‘focusing’ as a tool of resilience and articulating resilience as the individual’s ability and power to choose how to respond (6), self-reflection through an exploration about how the individual feels about themselves and their place in the world (7), activities that teach participants vocabulary and techniques that put distance between feelings and self (3), activities used to help listen and identify frustration, which is broken down into feelings vs. needs, and the relationship between them and how to identify them (8), building vocabulary of self-confidence (4), expressing feelings through clay, symbols, and the body (9), learning to identify very deep feelings and how to approach them so they can be talked about (10), techniques of extending empathy to the self (11), and methods for ensuring a balance of care for the self and care for the community (12).

Overall, Tarkiz uses far more visualization and somatic material than typical focusing programs. It introduces the idea of frustration as the experience of feelings and factors that can be parsed out and addressed (session 7), is structured so that intensely difficult feelings are accessed gradually once tools are in place and talks directly about the simultaneous need to care for the self and others (and balance these) (as in session 12). One of the most frequently noted sessions, and something clients and practitioners note as useful, is a session where participants identify a ‘safe space’ (s2). Unlike the presumption of a safe space for most therapeutic interventions, in Gaza there is no safety. Rather than falsely reassuring participants, they are asked to imagine a place of safety, either from their past or from imagination (moments, like a day at the beach with family, or a particular chair in their childhood home, a garden in the spring, etc.). It is to this place of safety that participants can retreat in order to listen to their feelings and analyze reactions to external events. Another session that clients often mark as a favorite uses the metaphor of the ‘green branch’ that bends when it is challenged, compared with the dry branch which snaps. Other sessions develop techniques of understanding, assessing, and vocalizing feelings, to the self and to loved ones, as well as learning to ‘listen’ with multiple senses to the feelings of others. Over the years PTC has developed an array of tools, materials, and even a handbook for clients to work through the sessions.

It is worth noting that this study appears to be the first academic work on an adaptation of Focusing in the Global South. While programs which implement or adapt Gendlin’s ideas on Focusing in the Global North have been studied for a variety of health matters, there has been no such data collection on Global South communities (22). Other communities in the Global South have adapted Gendlin’s ideas, such as Kosovo, Afghanistan, Ecuador, and El Salvador, however this work has only been documented informally and not to an academic standard, often failing to highlight the program’s true impact. While the International Focusing Foundation is more active in field training and grassroots initiatives, less academic attention has been paid to the success of such programs in confronting mental health issues beyond the Global North. A more detailed study of the nature and methods of the specific adaptations that PTC has made to the Focusing program remains to be carried out.

What PTC has already established, and continues to monitor, is the ability of Tarkiz to help its community. It does this by using a honed and verified intake and exit interview, where practitioners evaluate participants (20).

Before joining a Tarkiz program, clients are assessed on a locally adapted PTSD indicator list (20). Once the program is complete the assessment is given again. Clients who participated in the Tarkiz program reported a 78% reduction in the PTSD indicators (20). This of course is only one measure of success, and as we have seen, the use of PTSD as a metric of harm is not always –even when adapted—the most useful measure of the experience of harm and its redress. As such, our current study aims to evaluate the perceived success of the program and create a metric of success as defined by the clients and practitioners involved with the program. Indeed, as our results and discussion illuminate, it is not the reduction in specific symptoms which are reported as successful, but rather the ability to engage (or re-engage) with community and relate differently to ongoing harmful contexts present in Gaza.

This drive to re-define the terms of success when it comes to assessing and addressing mental health in a context of harm extended beyond the research question and into all aspects of how this study proceeded. From identifying a flexible and Arabic-language training program to gather qualitative data to taking the lead from PTC staff as they generated the questions about ‘definitions of success’ in Tarkiz, this research sought to be defined by and to capture local frameworks of mental health practice.

## Materials and methods

### Participants and recruitment

This qualitative study was conducted between May 2022 and March 2023. The interviewers were 11 PTC staff and former staffers who were trained in Arabic in qualitative methods via an adapted program developed by the Community Practitioner Research Program at the University of Birmingham via Zoom. During the training PTC participants were introduced to the purpose of the data collection (to understand the perceived effectiveness of Tarkiz, and the program’s adaptation over the years), and developed their own set of interview questions based on their own interpretation of how to assess perceived success. This training was carried out between May and July 2022, while interviews were conducted between September and November 2022. The training consisted of three key units that took participants through the research process from research design to data collection and analysis. The first unit consisted of online group training that covered designing and understanding social research, the second unit consisted of community research fieldwork and data collection with mentor support to learn about conducting social research, and the final unit consisted of data interpretation and report writing to learn about analyzing qualitative research findings. Data analysis was conducted between December 2022 and March 2023. Eleven interviewers carried out 30 interviews in total, 19 interviews were with clients and 11 were with practitioners (including but not limited to practitioners from the group being trained). Analysis reports were submitted by the interviewers for accreditation with the CPRP program. These reports informed analysis by the research team (23).

A qualitative approach was chosen as our method for this study because it enabled us to collect in-depth information about the program’s use within the community from multiple perspectives, and to learn as much from the generation of questions by the practitioners as from the collection of answers from both practitioners and clients.

### Study setting

The Palestine Trauma Centre provided the local hub for information gathering. Its offices are in a shared community space alongside another NGO, the Noor Almaarifa center, which provides services around children’s education. PTC offices are within this building in a separate area. The shared building is in the Nuseirat area, built up around the Nuseirat refugee camp. The Nuseirat area is located in the middle of the Gaza Strip, and prior to the October 2023 violence, there were approximately 85,000 Palestinian refugees in the camp registered with the UNRWA and it was composed of multiple school buildings, health centers, and relief and social services offices (24).

Note that at the time of writing, the offices of PTC are not in use. They were badly damaged in October 2023, and as of January 2024 most of the staff has been displaced. Programs are occasionally run in locations of refuge when resources permit.

### Recruitment and participants

The study population consisted of staff and clients at the Palestine Trauma Centre in Gaza. Dr. Mohamed Altawil invited all practicing staff and staff who had previously worked with the Tarkiz program to participate in the project. Initially 13 joined, but due to time constraints only the remaining 11 staff from the PTC received training. These staff then conducted interviews with 11 practitioners and 19 clients from the PTC. Interviewees, both practitioners and previous clients of the Tarkiz program, were recruited through PTC staff and community word of mouth.

### Data collection

Two research assistants from the CPRP at the University of Birmingham adapted the CPRP’s novel training program, which trains community members and practitioners in social research methods and supports them to identify and address social issues at a practice and policy level, to fit the context of the PTC. This program has been used with a wide range of marginalized communities to address multiple social problems, such as the resettlement of refugees and unaccompanied asylum-seeking children, migrant maternity and mental health, youth homelessness, and young offenders’ recidivism rates. With this experience in mind, the researchers believed that the program would have great potential to develop a model of research coproduction to support the PTC’s work in Gaza.

Once the program had been adapted into Arabic, it was delivered over Zoom via four units. The first three units took place between May and July 2022, while the last took place in small Zoom groups in September 2022. After a first session on basics and a brief overview of the rationale of the project, sessions were spent developing interview guides for clients and practitioners. While CPRP trainers (who were also given a broad brief of the research agenda) guided question creation for answerability, participants developed their own parameters for success and what might help or hinder that success. Their questions were informed by debates about developing the program that were local to the staff: for example they had received some feedback about the mixed-gender composition of the program, and so included questions about the suitability of the diverse groups (only one respondent expressed a desire for same-sex groups), and whether this might make groups more or less successful. In general, success for PTC staff was defined by whether clients used tools in everyday life, whether and how much they were comfortable taking the sessions, or if tools were shared with the client’s wider community. When making questions for practitioner interviews, PTC staff were primarily concerned about capturing information on the types and methods of change introduced by a practitioner to accommodate needs in the community, if/how they observed an increase in personal and community resilience, and to capture recommendations for possible change.

Once the training had been completed, PTC staff (11 in total) generated a topic guide with questions to ask both other staff and clients at the PTC based on a brief provided to the research assistants from the CPRP. This iterative process resulted in two sets of interviews and two topic guides, one aimed at clients of the PTC who had previously participated in the Tarkiz program, and the other aimed at both current and previous practitioners who had administered the program. The 11 trained PTC staff conducted the semi-structured in-depth interviews (IDIs) with practitioners and previous clients of the PTC in September to November 2022. IDIs were audio recorded and transcribed in Arabic by the PTC staff.

### Data analysis

We utilized qualitative thematic analysis to explore the nature of the perceived subjective experience of the Tarkiz Program. We analyzed the qualtative data in accordance with Braun and Clarke’s method (24). Inductive coding, a data-driven qualitative coding approach, involves interpreting the data without preconceived analytical frameworks, where a conceptual model is iteratively developed from the emerging themes that surface from the raw data (25).

The lead researchers read through all transcripts several times to familiarize themselves with the data while identifying key quotes. Applying inductive coding, three members of the researcher team coded the entire dataset and cross-referenced their codes. This involved generating the initial codes from the interview data and utilizing analytic process memos and group discussions to interpret the meaning of codes. Disagreements and discrepancies were addressed through ongoing discussions until a consensus was achieved. A codebook was then developed based on the coding of the data. This was discussed among the research team and final codes were agreed upon through group discussion.

**Figure 1:**
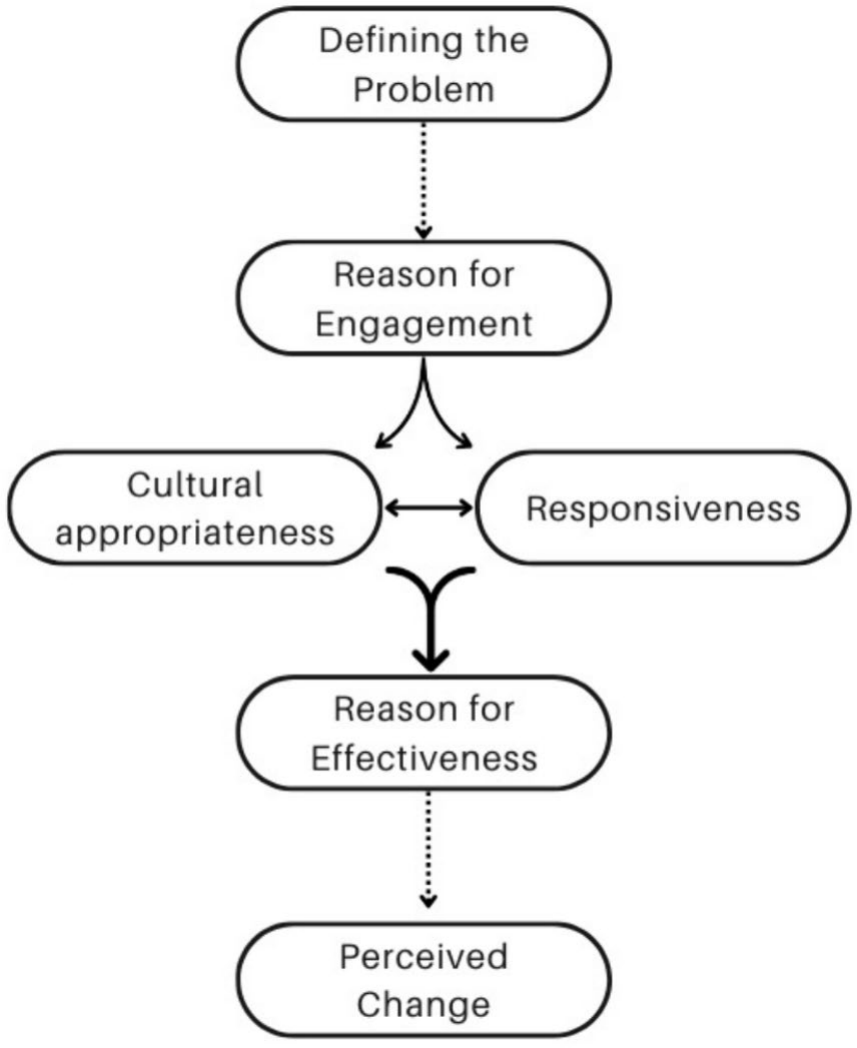
Relationship between themes.

Themes were established in an iterative process in which one researcher extracted themes from the data and two other researchers confirmed or disputed their choices. To deepen the analytic process, coders then discussed the preliminary emerging thematic groupings of the codes, ensuring the coherence of each theme and alignment with the overarching meanings in the dataset. The research team then developed a thematic map based on the key themes that emerged (see Figure 1 in Findings).

Adherence to best practice guidelines for qualitative research from the Critical Appraisal Skills Program guided both the data collection and analysis processes (26, 27). The research team documented the full process of thematic analysis, incorporating analytic process memos and reflective notes to establish an audit trail, to bolster the trustworthiness and credibility of the findings.

### Ethical considerations

This study received approval from the University of Birmingham ethics committee. The project was conceived and implemented in partnership with Dr. Mohamed Altawil, the founder of the Palestine Trauma Center, as part of a UK government funded research network (Rights for Time/ R4T) (28). Involved from the project’s inception, PTC and four other stakeholders from the global south were also key in developing the project’s ethical protocols, with which this work is in line. Informed consent was obtained from all study respondents, and confidentiality and privacy were prioritized during all phases of the study. All data collected from this research was anonymized and kept in secure data storage within the University of Birmingham. Because PTC is not affiliated with a university in Gaza, no secondary ethics approval was sought in Palestine, though at all stages project parameters were discussed with PTC leadership.

## Results

### Results of thematic analysis

Four main themes emerged from the thematic analysis of the qualitative interview data with both the practitioners and the clients. These themes were: (1) initial reason for engagement, (2) reasons for effectiveness, (3) cultural appropriateness, and (4) perceived change. The relationship between these themes can be seen in Figure 1. The total list of themes, subthemes, and codes is below in Table 1.

**Table 1:** Themes, subthemes, and codes.

	Theme	Subtheme	Code
Defining the problem	Reasons for engagement	Individual reasons	Multiple traumatic events
			Varied symptoms
			Addressing issues
		Referral	Trusted source
	Reasons for effectiveness	Approach of program	Helpful materials/practices
		Group dynamics	Group preferred
		Practicality	Physical accessibility
			Intellectual accessibility
		Frequency	Want more
	Cultural appropriateness	Mechanisms	Community focus
			Intergenerational/cross sectional
			Shared trauma/experience
			Gender
			Religion
			Calming techniques
		Requests for change	More diversity
		Confidentiality	Confidentiality
		Approach of practitioner	Practitioner sensitivity
		Wider context	Positive comparison
			Comparison
			First program used
	Perceived change	Community	Relationship with family
			Improved relationships
		Notable improvements	Emotional growth
			Coping mechanisms
		Application in real world	Used with others
			Referred others

Within each theme were numerous subthemes and codes. Theme 1, reasons for engagement, encapsulated the ideas discussed by the clients about what made them participate and retain participation with the Tarkiz program. This theme was split into the subtheme individual reasons and referral, and then split further into codes. The second theme, reasons that the program was effective, included the program’s approach, group dynamics, practical aspects of the program, such as physical and intellectual accessibility, and the frequency of the program. The third theme identified from our data was cultural appropriateness of the program, and was split into the subthemes of mechanism, or what it was about the program that felt culturally appropriate to the participants, requests for change, or aspects of the program participants thought may have made it more culturally appropriate, confidentiality as an aspect of cultural appropriateness, the approach of the practitioner, and how the program fit into the wider context of the participants’ situations. Finally, perceived change was identified as a theme in the data, which included how participants felt the program had helped them in their community, notable personal improvements they had noticed, and how they had applied what they learned in the program in the real world.

#### Initial reason for engagement

One of the themes coded in the transcripts was ‘initial reason for engagement,’ or what may have caused a client to seek out services at the PTC. This theme was split into two subthemes, ‘referral’ and ‘individual reasons,’ which were then split into the codes ‘addressing issues,’ ‘varied symptoms’, ‘multiple traumatic events,’ and ‘trusted source.’ Of the 18 quotes coded under the ‘engagement’ themes, two were about multiple traumatic events, three were about varied symptoms, three were about trusted sources for referral, and ten were about addressing specific issues.

Multiple traumatic events were mentioned as a reason why one might seek help from the PTC. For example, a 24 year old male client stated: ‘And I formed an idea and an expectation about the focus program that it can be a solution to my problem that I am going through in my life where I was exposed to multiple traumatic events and I found that this program is the right one to help me solve these problems.’ Varied symptoms were also mentioned by clients as the the issue that made them seek help, such as those mentioned by a 24 year old male client who stated: “I was going through behavioral problems, a sleep problem, and I remember the events as if they were passing in front of me, I have nightmares, I felt that my behaviors changed, I started to sleep a lot and I startle from anything. Even traumatic events, I started avoiding memories of places. These symptoms came to me after I was exposed to traumatic events in the last war in the month of May.” Finally, some clients had specific issues they wanted to address, such as a 37-year-old female client who stated: ‘I was suffering from extreme fear.’

With regards to ‘referral’ the researchers found that several participants spoke about being referred to the program by a trusted source. For example, a 47-year-old female client said: “A call from Basma Amal center tells me there is a psychological support course from Palestine center.” Responses about referral were split between either mentioning a trusted organization that had referred an individual to the center (16%) or self-referral wherein clients were hoping to address specific issues.

#### Reasons for effectiveness

The second theme that emerged from the data was the reasons why participants found the program effective. It was split into several subthemes: (1) the approach of the program, (2) group dynamics, (5) practicality, and (6) frequency. Of the 75 quotes coded under the ‘reasons for effectiveness theme,’ 41 were coded as ‘helpful materials/practices,’ nine as ‘physical accessibility,’ five as ‘intellectual accessibility,’ 15 as ‘group preferred,’ and five as ‘want more.’ Approach of the program included helpful materials or practices, for example a 33-year-old female practitioner noted that the program was helpful because it progressed with the needs of the clients: “Development is a continuous process, especially in psychological phenomena, the program is continuously evolving because it focuses on people’s needs.” Group settings were preferable to one-on-one sessions for some respondents. For example, a 25-year-old female client noted ‘Working with the group is better as we were benefiting from each other’s experiences, and I did not wish in any session to be an individual beneficiary.’ Practicality included physical and intellectual accessibility as another 25-year-old female client noted “The time of the sessions was suitable for everyone, the place was quiet, the place was in the middle of the area and easy to reach” and a 50+ year old male client who noted “It delivers information in a good, interesting and smooth way, especially to simple people, including me.” Frequency was mentioned by participants who wanted more sessions as the following quote from a 37-year-old female client indicated: “My recommendations are more sessions like this, and I will support everyone I know to take such sessions because they changed my life a lot.”

#### Cultural appropriateness

The third theme that emerged from the data was whether the participants felt the program was culturally appropriate to them. This theme was our largest, with many subthemes and codes falling under the umbrella of cultural appropriateness. Cultural appropriateness was thus divided into the following subthemes: (1) mechanisms, (2) requests for change, (5) confidentiality, (6) the approach of the practitioner, and (7) wider context. Cultural appropriateness was our most frequently identified theme, with 89 quotes being identified as relating to cultural appropriateness. Of these 89, 10 were coded as ‘community focus,’ three as ‘intergenerational,’ one as ‘shared trauma/experience,’ six as ‘gender,’ 10 as ‘calming techniques,’ one as ‘more diversity,’ 12 as ‘confidentiality,’ 33 as ‘practitioner sensitivity,’ two as ‘religion,’ one as ‘positive comparison,’ eight as ‘comparison,’ and two as ‘first program used.’

The subtheme of mechanisms was further divided into codes, including community focus, intergenerational/cross sectional, shared trauma/experience, gender, religion, and calming techniques. In discussing the importance of community focus, a 50+ year-old male client stated: ‘We are a group of mukhtars, notables and reformers who brought us together to participate in psychological support sessions and we all work for reform among people.’ A 30-year-old female client who was mentioning how she appreciated intergenerational/cross sectional approach of the program noted, ‘The group contains women of different ages and different ideas and backgrounds, but the program made us close together and we were one family.’ Shared trauma/experience was also an important feature of the program, as noted by a 29-year-old female client who said: ‘They needed sessions after the wars on the Gaza Strip and needed to be discharged, as well as benefited from the release of family and social problems to the extent that they felt that the topics spoke about them in particular.’ Gender was also listed as a helpful mechanism, as illustrated by a 37-year-old female client who said: ‘We are all females, and the age group was close.’ Religion was an especially important aspect of the program’s success, as many participants noted how important their faith was to them. One practitioner stated: ‘Indeed, the Tarkiz program took into account the cultural characteristics of the society in Gaza, and this was felt during the self-expression session through symbols and images, which touched upon the culture of the Palestinian society in general and the Gazan society in particular because the session contains Quranic verses and popular proverbs, as well as symbols and images, and the presence of these activities in the sessions was good.’ Another practitioner also discussed the importance of religion: ‘The challenge was whether this program suits our culture, being developed in a European environment. Indeed, it suited our culture, and this is the secret of its success. There are activities such as Quranic cards, where there was an intense interaction with the activity because of the fact that the Quranic verses reflected their feelings, and this has a positive impact’ (practitioner – 37, F).

The subtheme of calming techniques referred to the participants’ application of techniques learned through the Tarkiz program in their everyday lives. Examples of these techniques included the ‘safe space and green branch’ technique, as noted by a 52-year-old male client: “during the safe space session we talked about Qur’anic verses and asking for forgiveness from Allah. and we talked about people we felt safe with and how to protect them.’ Another client noted, ‘With this activity I felt something tangible that helped me differentiate between the green branch and the dry branch, and how the green branch gives hope and we, thanks to God have hope in God’s will and we have resilience (sumud, also, steadfastness)’ (client – 35, F). Both quotes illustrate how sometimes multiple codes could be applied to the same quote, as both mentioned calming techniques and religion.

Another subtheme under the cultural appropriateness theme was requests for change, which included a request for more diversity as noted by a 40-year-old female client: ‘Expanding with other groups and supporting those in need such as divorced and widowed women.’ Confidentiality was another important theme to participants, as indicated by a 50+ year old male client who stated: ‘It takes into account confidentiality and privacy, and we used to talk comfortably and show problems that happened with us and with people special problems and talk about them freely and feel that it was confidential without fear in order to shed light on them.’ The subtheme of approach of practitioner included ‘practitioner sensitivity’ a as noted by a client who, answering a question about the gender/age/status of the practitioners, noted: “Our relationship with the specialist was excellent, she came to deliver psychological support. She was like a sister.”

The subtheme of wider context included positive comparison, for example a 37-year-old female client noted: “The program was more accurate and deeper, and its effect was stronger” and a 58-year-old male client said: ‘I benefited from the Center for Democracy and Conflict Resolution in how to modify behaviors.’

#### Perceived change

This theme pertained to the results/consequences participants noticed from engaging with the program. It was split into three subthemes (a) community results/consequences, (b) notable improvements, and (c) application in the real world. Of the 64 quotes identified as a part of this theme, 19 were coded under ‘emotional growth,’ 22 under ‘coping mechanisms,’ two under ‘improved relationships,’ seven under ‘relationship with family,’ 12 under ‘used with others,’ and two under ‘referred others.’

The community results/ consequences subtheme included remarks around the improved relationship with family and relationships overall. One 24-year-old male client described the change in his relationship with family after taking the Tarkiz program: “Before I did not even know how to communicate with myself, now I have become better at listening to others, my relations with other have improved, I became a better listener to my mother, my father, my brother, and my friends. After the sessions, relationships that were tepid grew stronger.” Another client,35-year-old female stated: “[I benefitted] to the extent that I changed in how I treated my children, even those at home noticed the change.”

The notable improvements subtheme included remarks on personal emotional growth and the development of coping mechanisms that they continue to employ. An example of notable improvements was emotional growth as seen in this quote from a male client: “A more comprehensive and general view of the future and how we deal with these pressures.” An example of coping mechanisms comes from a male Mukhtar [local leader] who said: “By explaining how to manage feelings of anger, how to observe and deal calmly with anger, how to take a deep breath and notice where the anger is to relieve the pressure; this helped me and taught me patience and long-mindedness and all thoughts became good” A practitioner also noted that Tarkiz helped specifically with “Personal and everyday situations, where it changed their lifestyles, modified their thoughts, and also taught them some skills that enable them to adapt to life.”

The ‘application in the real-world’ subtheme included skills from Tarkiz being used with others or the participants referring others to engage with the program. For example, a 37-year-old female client stated that after her sessions, she would implement the activity at home with her children: “When I played with clay [at the session], I would then get my kids play with clay and when we did the activity of the teddy bear, I went home and had my kids talk to the teddy bear like we did.”

## Discussion

### Principal findings

The thematic analysis led to the development of four main themes, each of which were related to two meta-themes. Nearly all themes and codes in some way related to ‘perceived change’ which initially seemed obvious given the scope of the research, but also interesting was that many codes were also relevant to what was eventually defined as ‘defining the problem.’ This section will look at these two ‘meta themes,’ their relationship to each other, and how they were determined by the four main themes discussed above. The ideas are highly interrelated and interdependent, as illustrated in Figure 1. It is the whole set of themes and specifically the way they are related that we see as ultimately driving perceived effectiveness and indeed perceived change.

### Defining the problem - community as location of harm

The community of Nusseirat engaged in the Tarkiz program because it defined mental healthcare in a way that was recognized as valid and useful by the individuals it served. Reading the relationship between the themes, we can say that PTC defines mental healthcare as a community issue and locates the harm that it redresses at the level of community. In other words, Tarkiz understands that psychological harm is being experienced by the community and sees individuals as agents of that community. Ultimately, it sees that the harm experienced by the individual is not about the individual, but indeed takes place in the context of settler colonialism and occupation, where the legacies of harm and the ongoing harms specifically target the community. We see this most painfully at the present moment, where international courts are considering charges of genocide against Israel, a crime that specifically targets a group–Palestinians–en masse. Tarkiz’ definition of harm and approach to redress can be evidenced in some way across all the themes.

Most obviously, we see a definition of harm as something that targets community through “Reasons for Effectiveness” where descriptions of the ‘appropriateness’ of the approach of the program are all geared toward community. From universal accessibility (geographically and intellectually) to the program’s creation of group dynamics and long-lasting relationships, those interviewed noted that the sessions responded to the very practical considerations of life in a vulnerable area. These themes essentially fell into two categories: the accessibility of Tarkiz for a community-focused population, and the ability of all community members to engage with the method. As many people noted that the sessions were easy to get to and at appropriate times as did that the materials were particularly helpful. Those who noted group dynamics spoke about the creation of a community within the Tarkiz sessions, and the successful navigation by practitioners and organizers of often-delicate social hierarchies and customs around gender, age, and class. This is in part a feature of the ‘Focusing’ program that Tarkiz is built from, but also crucially because the practitioners and participants have all experienced the same multiple traumatic events that are being addressed and discussed in the sessions. Not only did this provide a safe space, but it modeled and replicated the kind of strong community that Tarkiz seeks to build; one that mirrors the community that participants seek. This was referenced not just as a reason that individuals felt comfortable engaging, but as a reason that it was particularly important for people in Gaza. The sense of strengthening a family and giving tools for the individual to engage in community was clearly seen as a key component of the work. This was particularly clear through interviews conducted with local mukhtars [local leaders], not only because the interviewees understood it as totally logical and acceptable that they as senior figures and role models would enroll in a mental wellness program, but because they saw the tools of Tarkiz as something that would help them lead and advise well. More broadly and given the context of stigma around mental healthcare in Gaza, having local leaders promote the program (and tell others that they have taken it), makes the service more accessible.

Speaking powerfully to the emphasis on harm as something that the individual experiences via the community (where the community is where harm is located) were comments around the materials used in the Tarkiz sessions. Notable here was the reference to somatic materials, from the ‘green branch’ used to demonstrate resilience to the activities that used clay or colored pencils to indicate where in the body a feeling resided, or the activity using a stuffed animal that helped to externalize feelings; these were the sessions that focused on individual agency within a problematic context, and externalization of harm; as something that is not a part of the individual but something they are experiencing. This particular construction of mental health and understanding of harm can also clearly be seen in the interviewees’ responses around ‘Cultural Appropriateness.’ The fact that Tarkiz has a community focus was of primary importance. Here we see an elaboration of notes around group dynamics, specifically how they mirror (in some ways) social dynamics to make the sessions acceptable/accessible. We see in ‘perceived effectiveness’ later how this setup also seems to make it easier to transfer skills learned in Tarkiz to family and community outside of the group. This transferal of skills not only empowers the individual, but was remarked on in many cases as a way to connect and repair broken bonds between individuals and their community. This could also be seen in comments about the utility of the calming techniques used in the sessions, which interviewees spoke at length about using in ‘daily life.’ We see that the mental wellness of the individual is discussed in relationship with the wider community. It is the individual who is trained in the tools for psychological wellbeing (the externalization of harm), but wellbeing is practiced through building links with community.

The definition of harm as something targeting the community (and experienced by the individual) also comes out clearly under the ‘Reasons for Engagement’ theme. First, for those who gave an ‘individual reason’ or ‘carried symptoms,’ or even the experience of ‘multiple traumatic events’ we saw repeated mention of an inability to carry out daily life tasks (taking care of family, engaging in community) as a ‘symptom’ that needed to be addressed. In other words, for many it was the breakdown in community relations/interactions that signaled a need for help. That others noted only that they had come to Tarkiz because of a referral only strengthens the importance of community, as PTC’s large network of relationships with other NGOs and healthcare institutions mean that individuals are coming through community-recognized pathways.

Not only is harm understood as happening to the community, but redress and repair also happen at the community level (through the individual, or ‘re-activating’ the individual within community). We see this through “Perceived Change,” where interviewees mention specifically repair to relationships and better communication with extended families. This accompanied mentions of personal and emotional growth, and also significantly a nearly universal mention that the program’s tools were used with others, or other members of their community were then referred to the program based on that personal experience.

Ultimately, harm and repair seem to be understood as an ongoing process. Continued engagement was directly related to the ongoing work of creating cultural appropriateness, which was part of the ‘responsiveness’ process. Being responsive and asking questions, being flexible and attuned to the needs of society also meant practitioners were listening for other potential shifts to the program that would make it more effective. It was all of this collectively that meant the program was perceived as effective, and this perceived effectiveness translated into perceived change.

### Science diplomacy

Science diplomacy involves using scientific collaboration and knowledge exchange as a means to build bridges, foster international cooperation, and address global challenges. It seeks to bridge global knowledge with local realities, and participatory research becomes a powerful conduit for ensuring that local voices are not just heard but actively contribute to shaping the research agenda. The involvement of local participants at various stages of the research is a dynamic and reciprocal process, crucial for identifying and prioritizing research questions that truly resonate with the community’s concerns. Their insights not only enrich the research agenda but also enhance the cultural relevance of the study, making it more attuned to the nuanced dynamics of the local context (29).

In the context of research, especially in sensitive and conflict-affected regions like Gaza, actively involving local participants is an essential strategy for several reasons. The active engagement of local participants in research, particularly through participatory methods, opens new questions and new avenues into what the ‘issues’ are to be solved, as well as how to best go about solving them. By creating an equitable partnership, and prioritizing the questions and concerns of local actors, not only is research culturally sensitive, but it gathers more nuanced information about what cultural sensitivity entails. This privileges local knowledge and gives the opportunity to include this knowledge into larger global debates. This approach recognizes the unique perspectives and values of the local community, and of the knowledge honed by mental health practitioners in areas like Gaza. Actively involving local participants empowers the community to contribute actively to the research process here, but also ensures that they have the research skills to continue work beyond the research partnership.

The active engagement of local participants is an instrumental force in the design of the research itself. Local perspectives bring a depth of understanding that is often unparalleled, guiding researchers in navigating the community’s values, traditions, and social structures. This collaboration is particularly vital in the context of mental health interventions, where cultural determinants significantly influence the effectiveness and acceptability of programs. Participatory research, when integrated into the framework of science diplomacy, becomes a holistic process where local participants are active contributors and co-creators of knowledge. Their involvement begins with identifying the best questions for data collection, and avenues into understanding a program, extends to identifying the best sources of information, and draws from their lived experiences and cultural insights to guide the research toward more accurate and comprehensive outcomes.

This diplomatic model transforms research into a collaborative effort that aligns with the principles of science diplomacy, emphasizing inclusivity, cultural relevance, and a shared commitment to addressing global challenges. Overall, the active involvement of local participants in research not only upholds ethical standards but also exemplifies a diplomatic approach that recognizes the interconnectedness of scientific endeavors on both local and global scales.

### Limitations and strengths

Understanding the perception of effectiveness of Tarkiz is only one part of a larger planned research project. Each phase has limitations but build on each other and build a large basis for further research given the challenging conditions in Gaza. Altawil had already found that Tarkiz was successful in significantly reducing the reported indicators for PTSD (20). Funding was secured via the Rights for Time network (28) in 2023 to finalize the Tarkiz manual in preparation for a Randomized Control Trial. This research work has halted, as staff and community endure genocide. The effectiveness of Tarkiz as it has been recorded here may never thus be completed. Since this will stand as part of a record of a mental health approach in Gaza ‘before’ the genocide, we must account for the limitations beyond those of qualitative data, which gives only personal perspectives, and relies on the thoughts and experiences of those who have been through the Tarkiz program. We must note also, of course, the possible importance of work understanding why individuals understand a program to be effective, given both the stigma and the urgency of mental health treatment in the coming years.

A strength and limitation of this study was that those conducting the qualitative research were trainees rather than professionals, meaning that the interviews were often short and stuck relatively closely to the script. This is because PTC staff were trained to develop the interview questions and carry out the interviews within their own communities. While the main questions of our study were therefore addressed, little additional information was obtained that may have provided further insight. Of course, by basing interviews on questions relevant to the community, and interviewing both practitioners and clients, we were able to gain insights into priorities that may have been invisible had the work been carried out by teams external to the community. Thus, we were able to broaden the understanding of the paper and the research team on what is important when it comes to the perceived success of a mental health program in Gaza. While we had hoped to collect more data around the history of adaptations made to the Tarkiz program over the years, this will have to wait for a later study. This will provide information for future culturally competent adaptations of PTSD interventions rooted in Western culture.

### Implication of the findings

The findings in this study would seem to suggest that adaptation of EuroAmerican treatment programs would benefit from cultural adaptation beyond language and cultural changes to the materials. Such an adaptation would seek to understand why potential users would seek help and how best to tailor programs to their specific needs. For example, the use of somatic devices which in this instance created a culturally relevant environment conducive to sharing and self-reflection.

Findings regarding the effectiveness of the program would suggest that an often-overlooked aspect of cultural adaptation is access to care, a critical component of healthcare provision in the Global South, especially in conflict areas. These practical considerations such as location, duration of the program, etc., be they physical or intellectual/emotional, could play a significant role in facilitating success of such programs.

Another key element in adaptation that was gleaned from the findings and would be relevant to Global South settings, is the importance of community engagement. The community as the unit of change, but also as an integral component in the mechanism of change. Another important factor highlighting the need to focus on the community, is that programs can depend heavily on leveraging existing social hierarchies and norms to establish trust and confidentiality, essential components of successful mental health programs. Furthermore, participants’ ability to apply program techniques outside of sessions demonstrated the seamless integration of Tarkiz into the community.

Our findings also point to the critical role that feedback mechanisms play in an adaptation process, as they ensure ongoing community acceptability and relevance, reinforcing commitment to continuous improvement and responsiveness to community needs.

By engaging in cross-cultural dialog and collaborative initiatives, science diplomacy can play a crucial role in bridging the gap between EuroAmerican treatment programs and the specific needs of communities in the Global South, particularly in conflict-affected areas. Collaborative projects involving researchers, mental health practitioners, and policymakers from diverse regions can provide a platform for shared learning, facilitating the adaptation of treatment programs beyond surface-level changes. Through these diplomatic efforts, practitioners can gain insights into the cultural determinants and practical considerations that impact access to mental health care in Global South settings. Furthermore, science diplomacy can contribute to building trust within communities, recognizing the importance of leveraging existing social hierarchies and norms to establish the crucial elements of trust and confidentiality. Emphasizing science diplomacy in mental health adaptation endeavors aligns with the broader goal of fostering international cooperation to address global health challenges, ensuring that cultural relevance and community engagement remain at the forefront of evolving mental health programs.

### Future research

Based on the implications of the findings outlined, future research could focus on several areas to further enhance the adaptation and effectiveness of mental health programs in settings like Gaza, impacted by ongoing conflict (context of harm).

#### Deeper cultural adaptation

Investigate strategies for cultural adaptation that extend beyond surface-level changes to materials and language. This could involve in-depth qualitative research to understand the underlying reasons why individuals seek help and how best to tailor programs to their specific cultural and contextual needs (a needs assessment tailored to the target community).

#### Enhancing access to care

part of that in-depth needs assessment would be to understand the practical considerations that impact access to mental health care in conflict-affected regions. This may include understanding physical/cultural/community/conflict factors impacting (barriers to) accessibility and participation. Understanding how these practical factors influence program success can inform the development of more accessible and effective interventions.

#### Community engagement strategies

Explore innovative approaches to community engagement as a key component of mental health program adaptation in the Global South. Investigate the role of the community not only as the unit of change but also as an active participant in the process, exploring ways to seamlessly integrate program techniques into community practices beyond formal sessions.

#### Feedback mechanisms and continuous improvement

Further investigate the importance of feedback mechanisms in the adaptation process and implementation of mental health Programs like Tarkiz. Explore different strategies for soliciting and incorporating feedback from program participants and community stakeholders to ensure ongoing acceptability and relevance. Research could also focus on the impact of continuous improvement efforts on program effectiveness and sustainability over time.

#### Long-term impact assessment

Conduct longitudinal studies to assess the long-term impact of adapted mental health programs in conflict-affected regions (Tarkiz and others). This could involve tracking changes in individual and community well-being over time to understand the sustained benefits of program participation (using tools developed for and by local communities). Longitudinal research can provide valuable insights into the lasting effects of mental health interventions and inform future adaptation efforts.

#### Science diplomacy

Science diplomacy can play an important role in advancing the research agenda outlined above, fostering collaboration and knowledge exchange across international borders. Establishing partnerships between researchers, mental health practitioners, and policymakers from different regions can bring diverse perspectives to the table, enriching the understanding of effective mental health interventions in conflict-affected settings. Collaborative projects that involve experts from both the Global North and the Global South can provide a platform for shared learning and innovative problem-solving. Furthermore, science diplomacy can facilitate the exchange of best practices, enabling the integration of successful strategies from various contexts. As researchers engage in cross-cultural dialog and collaborative initiatives, they contribute not only to the scientific community but also to the diplomatic effort of building bridges and fostering mutual understanding. This approach aligns with the principles of science diplomacy, emphasizing the importance of global cooperation in addressing complex challenges such as mental health in conflict zones.

By addressing these elements, researchers and practitioners can contribute to the ongoing refinement and improvement of mental health programs in conflict-affected regions, ultimately enhancing their effectiveness and impact on individual and community well-being.

## Conclusion

Overall, the findings from this research underscore the importance of culturally responsive and community-driven approaches to mental health intervention, particularly in conflict-affected regions like Gaza. The results point to the role of engagement, accessibility, cultural appropriateness, and feedback mechanisms in shaping the effectiveness and perceived impact of adapted programs like Tarkiz. Analysis showed how these mechanisms work together to identify the locus of harm differently than typical or western-normative interventions; shifting the perceived locus of harm from the individual to the community, where the individual was activated through Tarkiz to become an agent of community repair. This finding was only possible because of the locally-lead data collection and development of interview questions. In being asked to identify key factors in addressing research questions, local knowledge created the scaffolding for new parameters of perceived success.

Given the known limitations of implementing mental health interventions in contexts other than that which they were intended for, appropriately adapted programs, such as Tarkiz, provide crucial support to clients in a manner that is acceptable and applicable to them. By researching what makes programs like Tarkiz appropriate, we glean critical insights into the role and metrics of perceived success of mental health work in Gaza. As a long-term adaptation of an Anglo/European mental health approach, Tarkiz was perceived as effected because it embraces local perspectives, values, and practices. In understanding how these changes support perceived success, we get an expanded sense of what cultural adaptation looks like.

The international, equitable, and diplomatic nature of the research and the research team meant that local knowledge could be put into conversation with global questions. Work emphasized a collaborative approach, which was maintained not only through a research focus on cultural sensitivity in the adaptation of Tarkiz, but by employing these tools of science diplomacy to constantly adjust and interrogate the way the research unfolded. Partnerships were multidirectional, and adaptable, which are essential in global public health practices. Further, the capacity of Palestinian practitioners was expanded through training and participation so that further work might be done. All partners also now have the experience of a productive, responsive, and appropriate collaboration, which can serve as a model for future work for all members of the team.

## Data availability statement

The original contributions presented in the study are included in the article/supplementary material, further inquiries can be directed to the corresponding author.

## Ethics statement

The studies involving humans were approved by the University of Birmingham ethics committee. The studies were conducted in accordance with the local legislation and institutional requirements. The participants provided their written informed consent to participate in this study.

## Author contributions

SR: Writing – original draft, Writing – review & editing. RB: Writing – original draft, Writing – review & editing. NP: Writing – original draft, Writing – review & editing. AA: Data curation, Writing – original draft, Writing – review & editing. MA: Writing – original draft, Writing – review & editing. KE: Writing – original draft, Writing – review & editing.
